# Reported awareness of tobacco advertising and promotion in China compared to Thailand, Australia and the USA

**DOI:** 10.1136/tc.2008.027037

**Published:** 2009-03-29

**Authors:** L Li, H-H Yong, R Borland, G T Fong, M E Thompson, Y Jiang, Y Yang, B Sirirassamee, G Hastings, F Harris

**Affiliations:** 1 VicHealth Centre for Tobacco Control, The Cancer Council Victoria, Melbourne, Victoria, Australia; 2 Department of Psychology, University of Waterloo, Waterloo, Ontario, Canada; 3 Ontario Institute for Cancer Research, Toronto, Ontario, Canada; 4 Department of Statistics and Actuarial Science, University of Waterloo, Waterloo, Ontario, Canada; 5 Center for Chronic and Non-communicable Disease Control and Prevention, Chinese Center for Disease Control and Prevention, Beijing, China; 6 Institute for Population and Social Research, Mahidol University, Bangkok, Thailand; 7 Institute for Social Marketing, University of Stirling, Stirling, UK; 8 The Open University Business School, Milton Keynes, UK

## Abstract

**Background:**

China currently does not have comprehensive laws or regulations on tobacco advertising and promotion, although it ratified the World Health Organization (WHO) Framework Convention on Tobacco Control (FCTC) in October 2005 and promised to ban all tobacco advertising by January 2011. Much effort is needed to monitor the current situation of tobacco advertising and promotion in China.

**Objective:**

This study aims to examine levels of awareness of tobacco advertising and promotion among smokers in China as compared to other countries with different levels of restrictions.

**Methods:**

One developing country (Thailand) and two developed countries (Australia and the USA) were selected for comparison. All four countries are part of the International Tobacco Control (ITC) Policy Evaluation Survey project. Between 2005 and 2006, parallel ITC surveys were conducted among adult smokers (at least smoked weekly) in China (n = 4763), Thailand (n = 2000), Australia (n = 1767) and the USA (n = 1780). Unprompted and prompted recall of noticing tobacco advertising and promotion were measured.

**Results:**

Chinese respondents reported noticing tobacco advertisements in a range of channels and venues, with highest exposure levels on television (34.5%), billboards (33.4%) and in stores (29.2%). A quarter of respondents noticed tobacco sponsorships, and a high level of awareness of promotion was reported. Cross-country comparison reveals that overall reported awareness was significantly higher in China than in Thailand (particularly) and Australia, but lower than in the USA.

**Conclusions:**

There is a big gap between China and the better-performing countries such as Thailand and Australia regarding tobacco promotion restrictions. China needs to do more, including enhanced policy and more robust enforcement.

Banning tobacco advertising and promotion is an important part of the effort to curb the tobacco epidemic. Comprehensive advertising bans reduce tobacco consumption whereas partial bans have little or no effect.^1^^–^5 Article 13 of the World Health Organization (WHO) Framework Convention on Tobacco Control (FCTC) states that each party to the convention shall “undertake a comprehensive ban or,… restrict tobacco advertising, promotion and sponsorship on radio, television, print media and, as appropriate, other media, such as the internet”.^6^ Some countries have enacted comprehensive advertising bans and positive impacts have been reported.^1^ ^7^^–^10

The aim of this study was to compare smokers’ awareness of tobacco advertising and promotion in China with levels in Thailand and Australia (countries with strong policies) and with the USA (which has weak policies). This provides an indication of China’s relative progress towards eliminating this activity.

In China, it is estimated that over 350 million people smoke.^11^ Smoking kills some 1 million Chinese each year with economic costs in 2000 estimated at 5 billion US dollars.^12^ The Chinese government has made some efforts to implement laws and regulations to restrict tobacco advertising since the 1990s. The 1991 Tobacco Products Monopoly Law (Article 19) and the 1994 Advertisement Law (Article 18) ban direct tobacco advertisements on radio, television, newspapers and periodicals. The 1995 Tobacco Advertisement Management Regulations not only prohibit direct and disguised forms of advertisements in the above media (Articles 3 and 4), but also restrict competitions and programs connected with tobacco companies or their products brands (Article 8). However, there are gaps. There are no clear restrictions on outdoor and internet tobacco advertisements, and also little restrictions on tobacco company sponsorships. As a result, a range of marketing activities continue.^13^ ^14^

China ratified the WHO FCTC in October 2005, promising to ban all tobacco advertising by January 2011. Like China, Thailand and Australia have ratified the FCTC. The US has yet to ratify the FCTC.

In Thailand substantial tobacco control efforts have been made over the years, including laws and regulations designed to limit access to tobacco products, placing bans on displaying cigarettes and on various advertising, and enhancing pictorial health warnings on cigarette packets.^1^ ^8^ ^9^ ^15^ ^16^ The Tobacco Products Control Act 1992 comprehensively banned advertising and promotion and made most forms of promotional activities illegal. These restrictions on tobacco marketing have been reasonably well enforced, despite a reported increase in point-of-sale advertising and indirect marketing since 1997.^8^ ^9^ ^15^

Australia has a well known record on tobacco control, although smoking prevalence is still high among the Aboriginal population.^10^ ^17^ Considerable progress in banning advertising has been achieved since federal legislation banning direct cigarette advertising on television and radio came into effect with the Australian Broadcasting and Television Act Amendment ACT 1976. Advertising was banned in print media in 1993 and outdoors in 1996. Several states have banned point-of-sale advertising and have considerably limited the number of packs permitted to be displayed.^10^ ^17^^–^19 Sponsorships of sport and arts were also banned by 1996,^19^ but exemptions were allowed until 2006 for internationally significant events, most notably Formula 1 Grand Prix motor racing.

The US has fewer restrictions compared to Thailand and Australia. In response to the first Surgeon General’s Report on Smoking and Health, Congress enacted the Cigarette Labelling and Advertising Act in 1965, which required health warnings on all cigarette packages. The 1969 Federal Public Health Cigarette Smoking Act banned advertising of tobacco from television and radio. The 1998 Tobacco Master Settlement Agreement has restricted marketing to some extent.^20^ ^21^ However, the restrictions were not comprehensive, with many marketing channels open. As a result, the tobacco industry has taken advantage of this and expanded their marketing in areas where it is allowed. In 1999 the overall tobacco advertising expenditures in the US was $8.24 billion, an increase of 22.3% compared with 1998; spending in newspapers increased by 73%, magazines by 34.2% and direct mail by 63.8%.^21^ ^22^ According to the Federal Trade Commission, total cigarette advertising and promotional expenditures remained as high as $14.15 billion in 2004 and $13.11 billion in 2005.^23^

## METHODS

### Sampling design and procedures

The International Tobacco Control (ITC) China Survey is a prospective face to face cohort survey of adult smokers (at least weekly use) and non-smokers conducted between April and August 2006 in 6 cities (800 smokers and 200 non-smokers in each city: Beijing, Shenyang, Shanghai, Changsha, Guangzhou and Yinchuan). These cities were selected based on geographical representations and levels of economic development. Within each city there was a random sample selected using a stratified multistage design. In each of the 6 cities, 10 Jie Dao (street districts) were randomly selected at the first stage, with probability of selection proportional to the population size of the Jie Dao. Within each selected Jie Dao, two Ju Wei Hui (residential blocks) were selected, again using probability proportional to the population size. Within each selected Ju Wei Hui, a complete list of addresses of households was first compiled, and then a sample of 300 households were drawn from the list by simple random sampling without replacement. The enumerated 300 households were then randomly ordered and approached accordingly until 40 smokers and 10 non-smokers were surveyed. Because of low smoking prevalence among women, one male smoker and one female smoker from every selected household were surveyed whenever possible to increase the sample size for women. At most one non-smoker was interviewed per household. Where there was more than one person in a sampling category to choose from in a household, the next birthday method was used to select the individual to be interviewed.

The survey interviewers were trained by staff from local Centers for Disease Control. The average time to complete a survey was about 30 min for smokers and 10 min for non-smokers. Up to four visits to a household were made in order to interview the target person. The wave 1 cooperation rates range from 80% in Beijing and Guangzhou to 95% in Changsha. The response rates range from 39% in Yinchuan to 66% in Guangzhou. A total of 4763 smokers and 1259 non-smokers were included in the analysis. Additional information about the ITC China survey methodology and sampling is available at http://www.itcproject.org.

Thai participants came from the first wave of the ITC Southeast Asia (ITC-SEA) survey, which was conducted in January to February 2005. Respondents were selected based on a multistage cluster sampling procedure. The primary strata consisted of regions. Respondents were selected from Bangkok and two provinces in each of Thailand’s four regions. There was a secondary stratification into rural and urban regions within each province. Subdistricts and communities were selected within urban and rural districts, with probability proportional to population size, for a total of 125 sampling clusters of about 300 households in the whole country. Households were selected within each cluster using simple random sampling until the respondent quota (16 adult smokers) in each cluster was filled. In households with more than one eligible respondent per quota cell, respondents were randomly selected by using a Kish Grid. A total of 2000 smokers were surveyed through face to face interview. A more detailed description of the ITC-SEA study can be found in Yong *et al* 2008.^9^

Australia and the USA are part of the ITC Four Country Survey, which has been running annually since 2002. Participants used here were 1767 Australians and 1780 US smokers surveyed in wave 5, conducted from September to December 2006. They were interviewed over the telephone and were recruited by probability sampling methods (random digit dialling methods from list-assisted phone numbers). A detailed description of the ITC conceptual framework, methodology and survey rates has been reported by Fong *et al* (2006) and Thompson *et al* (2006),^24^ ^25^ and more detail is available at http://www.itcproject.org.

### Measures

In addition to demographic and smoking related information, relevant questions measuring awareness of tobacco advertising and promotional activities were included. At the beginning of “advertising” section of the survey the respondents were asked about the overall salience of pro-smoking cues (unprompted recall): “In the last 6 months, how often have you noticed things that are designed to encourage smoking or which make you think about smoking?”. The smokers were then prompted to recall if they had noticed advertisements in a range of specific locations or media, including five common to all countries: on television, radio, posters/billboards, newspapers and in stores. The measures used from this were either a total of the five where advertising was seen, or a binary, seen-any variable. Noticing at point of sale was also measured in all countries. There were also questions about awareness of sports and arts sponsorships (with a combined measure for noticing either); and an index created from responses to whether a respondent reported noticing any of the following four types of promotion: free samples of cigarettes, gifts/discounts, branded clothing or competition. Two overall indices of awareness across all three types of marketing were computed: “total noticing advertising, sponsorship and promotion in any channel” and “total number of channels of noticing”. In addition, smokers were asked to indicate whether they agree with the following statement: “Tobacco companies should be allowed to advertise and promote cigarettes as they please”. The survey questions were carefully translated and back-translated and checked to ensure conceptual identity of questions across languages.

### Data analyses

The analyses were conducted on weighted data using SPSS V. 14.0 (SPSS, Chicago, Illinois, USA). Differences between samples were assessed using Pearson χ^2^ tests and logistic regression models (for categorical variables) and Kruskal–Wallis test for count variables. An α level of p<0.05 was used for all statistical tests.

## RESULTS

### Demographic and smoking related characteristics

Nearly all the Chinese sample (95.2%) were of Han ethnicity. Other sample characteristics are shown in table 1. The respondents were overwhelmingly male, and smoked only factory-made cigarettes (93.8%).

**Table 1:** Demographic and smoking-related characteristics of the smokers, by country

	China (%) (n = 4763)	Thailand (%) (n = 2000)	Australia (%) (n = 1767)	USA (%) (n = 1780)
Gender (male)	95.9	95.5	54.0	53.9
Age:				
18–24	1.9	6.7	11.5	13.2
25–39	18.0	24.5	37.0	31.4
40–54	47.6	41.1	34.5	36.9
55+	32.4	27.7	16.9	18.5
Education:*				
Low	13.7	75.0	62.7	45.9
Moderate	66.3	17.5	22.8	36.6
High	20.0	7.5	14.5	17.6
Income:				
Low	20.8	54.6	28.1	36.9
Moderate	48.8	30.4	33.8	34.6
High	30.4	15.0	38.1	28.5
Cigarettes per day:				
0–10	35.0	55.7	27.8	31.7
11–20	48.7	36.9	42.4	46.3
21+	16.3	7.4	29.8	22.0
Type of cigarettes smoked:				
Factory-made only	93.8	41.8	74.8	90.0
Roll your own only	1.1	32.9	11.8	1.5
Both	5.1	25.3	13.4	8.4

Relative levels were used for education and income across countries.

*Education in China and Thailand: low, no schooling/elementary; moderate, middle school (secondary); high, tertiary (college or higher). Education in Australia and the US: low levels of education were considered to be completed high school or less; moderate levels were considered to be technical/trade/some university (no degree in Australia, and community college/trade/technical school/some university (no degree) in the US; high levels were those who completed university and/or postgraduate degrees.

As in China, the vast majority of the Thai sample were men, while the proportion of male smokers was about 54% in Australia and the US, reflecting the low smoking prevalence among women in China and Thailand. Young people aged 18–24 were notably under-represented in the Chinese and the Thai samples. The age compositions of the Australian and American samples were comparable. Due to the differences in economic development and educational systems across the four countries, only relative levels of income and education were used. The Thai sample had more roll your own smokers especially compared to China and the USA, and the Thais also smoked fewer cigarettes per day.

### Reported awareness of advertising and promotional activities

Without prompting, overall 40.3% of Chinese smokers reported noticing things that were designed to encourage smoking at least once in a while in the last 6 months (table 2). This was significantly higher than that in Thailand (20.2%), Australia (18.9%) and the US (35.5%) (p<0.001). Unprompted recall was higher than for non-smokers (table 3).

**Table 2:** Awareness of tobacco marketing activities among Chinese smokers (n = 4763), by age

	Total (%) (n = 4763)	18–29 (%) (n = 242)	30–44 (%) (n = 1405)	45–59 (%) (n = 2044)	60+ (%) (n = 1072)
Overall salience					
Noticed things that encourage smoking in the last 6 months:*					
Never	57.3	55.0	55.7	58.4	57.5
Once in a while	23.2	30.6	26.1	21.5	21.0
Often	17.1	13.6	16.7	17.2	18.2
Advertisements					
Noticed tobacco adverts (yes):					
On television	34.5	50.6	37.3	31.6	32.7
On radio	14.2	15.4	13.6	13.1	16.5
On posters	20.7	38.4	25.6	17.2	16.8
On billboards	33.4	50.4	39.4	32.7	23.2
In newspapers, magazines	19.1	24.8	18.8	18.5	19.4
In cinema	6.0	10.8	7.6	5.0	4.7
Over the internet	3.8	16.1	5.6	2.5	1.1
At workplace	11.1	19.0	13.0	10.5	8.0
On transport vehicles, stations	18.0	26.6	19.8	16.3	16.9
In cafeterias/tea houses	13.7	29.0	15.7	12.2	10.3
In discos, karaoke lounges	10.4	30.2	15.0	7.6	5.2
At point of sale					
in stores	29.2	56.2	37.9	26.1	17.7
around street vendors	20.3	36.0	27.1	17.5	13.1
Any venue above	62.6	79.6	71.4	58.8	54.6
Mean (SEM) number of venues noticed tobacco adverts	2.34 (0.04)	4.03 (0.22)	2.76 (0.08)	2.10 (0.06)	1.86 (0.08)
Sponsorships:					
Seen/heard sports event sponsorship (yes)	26.0	49.2	34.4	23.6	14.5
Seen/heard arts event sponsorship (yes)	8.4	13.3	11.2	7.5	5.1
Any type of sponsorship	27.9	51.6	37.0	25.1	15.8
Promotional activities:					
Noticed/seen free samples of cigarettes (yes)	13.8	23.7	21.7	11.4	5.8
Special price offers for cigarettes (yes)	12.9	26.4	17.2	11.7	6.3
Gifts/discounts on other products (yes)	22.5	32.6	27.6	22.2	14.3
Clothing with cigarette brand name (yes)	11.4	32.6	15.3	9.6	4.8
Competitions linked to cigarettes (yes)	8.5	21.1	13.0	7.1	2.4
Any form of promotion	38.5	59.6	50.0	37.0	21.4
Total noticing advertising, sponsorship and promotion in any channel	75.6	87.7	84.4	73.9	64.7
Mean (SEM) overall number of channels of noticing advertising, sponsorship and promotion	3.38 (0.05)	6.02 (0.28)	4.16 (0.10)	3.03 (0.08)	2.39 (0.09)

Group differences for all the individual variables of interest are significant at p<0.01 level based on Pearson χ^2^ test.

*About 2.4% of respondents chose “Don’t know” option.

**Table 3:** Unprompted recall of noticing things that encourage smoking in the last 6 months among Chinese smokers and non-smokers*

	Smokers (n = 4763), n (%)	Non-smokers (n = 1259), n (%)
Never	2721 (57.3)	896 (71.3)
Once in a while	1103 (23.2)	231 (18.4)
Often	814 (17.1)	87 (6.9)
Don’t know/Cannot say	114 (2.4)	43 (3.4)

*Weighted data. There were 11 missing cases for smokers and 3 missing cases for non-smokers.

For prompted recall, total noticing advertising, sponsorship and promotion in any channel among Chinese smokers was 75.6% with an average of 3.4 (SEM 0.05, table 2) channels noticed, the most common being television (34.5%), billboards (33.4%) and at points of sale (29.2% in stores and 20.3% around street vendors).

Overall, the younger Chinese smokers were more likely to have noticed various marketing activities with the exception of radio where the aged 60 and older were just as likely to notice such activities as the 18–29-year-old group (table 2).

Compared to the other three countries in table 4, the total prompted recall proportion in China (73.4%) was significantly lower than that in the US (95.3%, adjusted odds ratios (AOR) = 9.32, 95% CI 7.19 to 12.07, p<0.001) but much higher than that in Thailand (22.4%, AOR = 0.10, 95% CI 0.09 to 0.12, p<0.001) and somewhat higher than Australia (60.3%, AOR = 0.63, 95% CI 0.54 to 0,73, p<0.001). A similar pattern was evident when using mean number of venues noticing advertisements: highest in the US (89.6%, mean 1.90), followed by China (58.9%, mean 1.34), then Australia (40.2%, mean 0.60) and the least among the Thai (14.5%, mean 0.22). However, when “noticed advertisements in stores” was examined, Australian respondents were more likely to notice advertisement inside the stores than their Chinese counterparts (33.0% vs 29.2%, AOR = 1.18, p<0.05), although still far less than US smokers (p<0.001).

**Table 4:** Comparison of awareness of tobacco marketing activities, by country

	China (%) (n = 4763)	Thailand (%) (n = 2000)	Australia (%) (n = 1766)	USA (%) (n = 1779)
Salience: noticed things that encourage smoking in last 6 months (At least once in a while)‡	40.3	20.2	18.9	35.5
Adjusted OR (95% CI)	Ref	0.33 (0.29 to 0.38)***	0.33 (0.28 to 0.39)***	0.74 (0.65 to 0.85)***
Noticed tobacco advertisements in any of the five media¶	58.9	14.5	40.2	89.6
Adjusted OR (95% CI)	Ref	0.11 (0.09 to 0.13)***	0.49 (0.43 to 0.56)***	6.73 (5.57 to 8.13)***
Mean (SEM) number of venues noticed tobacco adverts§	1.34 (0.02)	0.22 (0.02)	0.60 (0.02)	1.90 (0.03)
Noticed tobacco advertisements in stores (yes)	29.2	3.6	33.0	84.9
Adjusted OR (95% CI)	Ref	0.09 (0.07 to 0.11)***	1.18(1.02 to 1.37)*	15.07 (12.69 to 17.90)***
Noticed sports event sponsorship (yes)	26.0	3.5	21.6	22.1
Adjusted OR (95% CI)	Ref	0.13 (0.09 to 0.16)***	1.09 (0.93 to 1.28)	1.11(0.96 to 1.29)
Noticed arts event sponsorship (yes)	8.4	0.4	1.9	9.3
Adjusted OR (95% CI)	Ref	0.04 (0.02 to 0.09)***	0.26(0.18 to 0.38)***	1.30(1.04 to 1.64)*
Noticed any type of sponsorship	27.9	3.7	22.1	26.6
Adjusted OR (95% CI)	Ref	0.12 (0.09 to 0.15)***	1.01(0.86 to 1.18)	1.27 (1.10 to 1.48)**
Noticed special price offers for cigarettes (yes)	12.9	2.5	23.0	71.4
Adjusted OR (95% CI)	Ref	0.17 (0.12 to 0.22)***	1.74 (1.47 to 2.07)***	15.61 (13.35 to 18.27)***
Noticed any other form of promotion†† (excluding special price offers)	36.6	9.5	31.0	82.0
Adjusted OR (95% CI)	Ref	0.18 (0.15 to 0.21)***	0.74 (0.64 to 0.86)***	8.67 (7.38 to 10.19)***
Total noticing tobacco marketing in any channel	73.4	22.4	60.3	95.3
Adjusted OR (95% CI)	Ref	0.10 (0.09 to 0.12)***	0.63 (0.54 to 0.73)***	9.32 (7.19 to 12.07)***
Mean (SEM) overall number of channels of tobacco marketing§	2.38 (0.03)	0.38 (0.02)	1.22 (0.04)	3.92 (0.05)

All odds ratios (ORs) are adjusted for age, sex, education and income.

*Significant at p<0.05; **significant at p<0.01; ***significant at p<0.001; ‡this includes “very often”, “often” and “sometimes/once in a while”; ¶these five media were television, radio, posters/billboards, newspaper/magazines and stores (posters/billboards was a composite variable in Australia and the US); §significant country difference at p<0.001 based on Kruskal–Wallis Test; ††these forms include free samples of cigarettes, gifts/discounts on other products, clothing with cigarettes brand name and competitions linked to cigarettes.

Ref, reference value.

Awareness of sponsorship followed a similar pattern, about a quarter of Chinese smokers reported noticing sport sponsorship and 8.4% reported noticing arts sponsorship, similar levels to Australia (AOR = 1.01, p = 0.89), more than Thailand (AOR = 0.12, p<0.001), while American respondents were more likely to report sponsorship (AOR = 1.27, p<0.01), although not by as much as for other promotional forms.

Overall, 38.5% of Chinese smokers reported noticing any of the five types of promotion, with just over a fifth reported noticing free gifts or special discount offers on other products, and 13.8% reported noticing free samples of cigarettes (table 2). Again, American smokers were the most likely to be exposed to special price offers for cigarettes (71.4%, table 4), followed by respondents in Australia (23%), then Chinese (12.9%) and the least in Thailand (2.5%). For other forms of promotion, a similar pattern was found: US smokers were the most likely to be exposed (82%), with much lower proportions among the Chinese and Australian respondents (36.6% and 31.0%, respectively) and the least among the Thai sample (9.5%).

### Opinions regarding tobacco companies’ advertising and promotion

**Figure 1:**
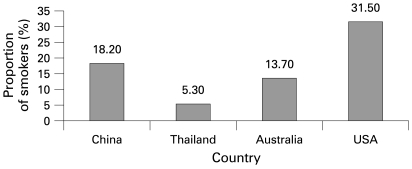
Proportion of smokers agreeing to allow tobacco companies to advertise and promote cigarettes as they please.

Figure 1 presents smokers’ attitudes towards tobacco companies’ advertising and promotional activities. Overall, only 18.2% of the Chinese smokers agreed or strongly agreed that tobacco companies should be allowed to advertise and promote cigarettes as they please. The endorsement rates were considerably lower among smokers in Thailand and Australia (5.3% and 13.7% respectively), but markedly higher among smokers in the US (31.5%).

## DISCUSSION

This is the first study of which we are aware to assess the extent of tobacco advertising and promotion in China. The results show that most Chinese smokers in the six selected cities frequently reported noticing tobacco marketing activities in various forms and channels, particularly in the media and at points of sale. In addition, there were considerable levels of reported exposure in some other venues, such as on public transport vehicles and at workplaces.

**What this paper adds**

- This paper is the first paper to document Chinese smokers’ reported awareness of tobacco advertising and promotion. It has identified a big gap between China (at least in the six selected cities) and the better-performing countries such as Thailand and Australia regarding effective tobacco promotion restrictions.
- This paper demonstrates the need for China to implement its promise to ban all tobacco advertising by 2011 and suggests that if it is to be maximally effective, it should eliminate existing loopholes and be accompanied by robust monitoring and enforcement.

The main limitation of this study is the use of respondent reports to provide information on exposure to marketing activities. Reported awareness is a function of the presence of the promotional activity, the respondent paying sufficient attention to it to remember it, and being able to retrieve it from memory when asked. While such measures are indirect and imprecise, they have some validity as it has been shown that at least some of these measures are sensitive to changes in policies within countries.^7^ The recall period is also notional, as most memories of advertisements do not come “time stamped” in memory, so timing will often be guessed. For example, more salient events may be recalled from before the given time window. Similarly, there is likely to be some misremembering of venues or media in which advertisements or other activity was reported. These measures should not be considered to be absolute levels of recall,^9^ let alone of actual promotional activity, merely as one that should provide reasonable ranking of exposure levels. If there is a systematic bias, it is more likely to be over-reporting in countries where the target advertisements are rare, as those that appear may have increased salience, and underreported where advertisements are common, as they will be more likely to be taken for granted. If this speculation is correct, then the real differences in promotional activities may be even greater than our data suggest.

Other problems are less likely to have had large effects. Since the methods used were not identical in all four countries, it remains possible that some of the differences found are at least partly due to such differences, but we think large effects are unlikely. In China the sample was not a nationally representative sample, unlike the three comparison countries, being restricted to six cities. Also, like Thailand, it under-represented young people. An urban only sample is likely to have higher awareness of forms of promotion that occur most often in towns and cities,^9^ while the under-representation of young people is likely to reduce estimate sizes as they were more likely to report promotions in most areas. It is also possible that there are effects as a function of mode of surveying (phone versus face to face), but we can think of no strong rationale for any large effect in either direction. Further, this cannot explain any of the differences with Thailand. Any net effect of these differences is likely to be small relative to the large country differences we typically found. That said, care should be taken in interpreting small significant effects, as these could be artefacts of these complex of factors. Particular care should be taken in interpreting demographic difference, where sensitivity to exposure could produce effects independent of actual exposure. We suspect that lower level of interest in tobacco promotions is probably why non-smokers report lower awareness.

It is possible that some of the age effects on noticing promotions are also a function of younger people being more likely to pay attention to, or remember things in their environments, rather than of any selective targeting of them. However, some of the differences found almost certainly reflect real differences in exposure, as the areas where the age differences were greatest (eg, internet, discos) are places where young people are more likely to be exposed. Further, there is evidence that the tobacco industry selectively targets youth in other countries,^1^ ^13^ ^20^ so it is credible that this also happens in China. While this study is not focusing on young people vulnerable to uptake, it is likely that they will also be reached through promotions in youth-focused venues, suggesting they might be a priority area for tightening restrictions. Point of sale was another area where younger smokers were much more likely to notice things. Here the explanation is less likely to be greater opportunity, but a greater interest in such material. Regardless of the explanation, it is of concern, as young people are priority group to protect from smoking. Comprehensive laws need to reach all these kinds of places.

Compared to the USA, where there are constitutional barriers to fully constraining tobacco promotion, China is doing well, but the comparisons with Australia and particularly Thailand show that there is great potential for improvement. Thai respondents consistently reported very low levels of exposure, either to advertising, sponsorship or to promotion, suggesting well enforced restrictions, as has been reported previously.^3^ ^8^ ^9^ Australian respondents also reported low awareness, but with higher than desirable levels in stores, sports sponsorship, as well as special price offers for cigarettes. This is consistent with earlier findings of Harris *et al*^7^ using the same survey tool 3 years earlier, and reflects gaps that have not yet been closed or, in the case of sponsorship, closed too recently for the memories to have dissipated. Sponsorship of the high profile Formula 1 Grand Prix ended only months before our survey in Australia. It is apparent that where promotion is allowed, or there are opportunities to get around existing laws, the tobacco companies will take advantage of it.

The Chinese government (and other governments) should be reassured by the high levels of support in all countries for restricting advertising. Presumably, smokers realise that smoking is dangerous and don’t want tobacco companies making it harder for them to quit. A recent review of the literature concluded that tobacco promotions encouraged smoking among non-users and increased consumption among tobacco users.^26^

China can learn a lot from the experiences of other countries, especially those with strong controls on tobacco promotion. What China should be aiming to achieve is a genuine comprehensive ban. Achieving it will require new policies that address the areas that are not covered by the existing laws and regulations, such as outdoor and internet tobacco advertisements, and advertising and promotional activities at workplaces and other public places. It also needs to focus on point of sale. At the same time, China needs to reinforce enforcement of all existing laws and regulations regarding tobacco advertising and promotion, as there are still disturbingly high levels of reported promotions from many of these areas.

In conclusion, there is no doubt that Chinese smokers report higher levels of tobacco promotion in their environments than Australian or particularly Thai smokers. China needs to do more to comprehensively eliminate tobacco promotion to fulfil its FCTC obligations. Models exist, especially in Thailand, of how to do this.
